# miR-125a-5p Regulates Osteogenic Differentiation of Human Adipose-Derived Mesenchymal Stem Cells under Oxidative Stress

**DOI:** 10.1155/2021/6684709

**Published:** 2021-06-07

**Authors:** Yongheng Ye, Quan Liu, Changzhao Li, Peiheng He

**Affiliations:** ^1^Department of Joint Surgery, The First Affiliated Hospital of Sun Yat-sen University, Guangzhou 510080, China; ^2^Department of Orthopaedic Surgery, The First People's Hospital of Nankang, Ganzhou 341400, China

## Abstract

Adipose-derived mesenchymal stem cells (ADSCs) are a well-recognized multilineage stem cell with vital clinical feasibility for tissue regeneration. Extensive evidence indicates that oxidative stress and microRNAs (miRNAs/miRs) play an important role in the osteoinduction of adipose-derived mesenchymal stem cells. In this study, we investigated the mechanism of miR-125a-5p in regulating the osteogenesis of human adipose-derived mesenchymal stem cells (hADSCs) under oxidative stress. The expression of miR-125a-5p lessened gradually during the osteogenic differentiation of hADSCs. Relative to the negative group, the expression levels of runt-related transcription factor 2 (RUNX2), alkaline phosphatase (ALP), osteocalcin (OCN), and osterix in the miR-125a-5p group were marked lower than those in the miR-125a-5p inhibitor group. The levels of p16, p21, p53, miR-125a-5p, and ROS during osteoinduction of hADSCs were assessed in vitro under oxidative stress and were observed to be upregulated. Further experiments showed that oxidative stress and miR-125a-5p together suppressed the expression of VEGF during osteogenic differentiation of hADSCs and that the inhibition of miR-125a-5p reversed the effect of oxidative stress. In short, our study indicated that miR-125a-5p is induced under oxidative stress and inhibits the expression of VEGF, leading to the reduction of osteogenic differentiation of hADSCs. Our outcomes showed that miR-125a-5p could be a potential clinical target for bone repairing.

## 1. Introduction

Bone loss caused by trauma, tumor, and age-related osteoporosis is a common problem in orthopedics. The balance of bone metabolism is a dynamic course influenced by osteoblasts, osteoclasts, and osteocytes. Oxidative stress, causing many reactive oxygen species (ROS) to form, plays an important role in the development of skeletal diseases [1–3]. The activity of oxidants has also been proven to enhance osteoclast differentiation [4]. With the need for more effective treatment, it is of great significance to find osteogenic methods for bone repair and regeneration.

Stem cells can differentiate into the bone, cartilage, and fat under the stimulation of certain factors, providing promising therapeutic applications with regard to the restoration of tissue defects [5–7]. Many researches have focused on efforts to utilize bone mesenchymal stem cells (BMSCs) for bone tissue engineering [8]. In addition, previous studies have proven that VEGF regulates adipocyte and osteoblast differentiation in mesenchymal stem cells [9]. Adipose-derived mesenchymal stem cells (ADSCs) as seed cells are more readily available to that of BMSCs, but their osteogenic differentiation ability is limited [10]. Aging is the main factor leading to decreased activity and repair ability of mesenchymal stem cells [11]. Oxidative stress is one of the main causes of aging. There is evidence to show that oxidative stress suppresses osteogenic differentiation and weakens the stemness of MSCs, which causes bone formation defects [12–14]. In addition, oxidative stress can induce apoptosis, senescence, and death of MSCs [15]. Therefore, clarifying the regulating mechanisms of osteogenic differentiation of ADSCs is helpful for identifying their possible therapeutic applications in bone repair.

MicroRNA (miRNA) is a single stranded RNA molecule consisting of about 18-22 nucleotides, which play a key role in cell apoptosis, proliferation, tumorigenesis, and other physiological and pathological processes [16]. Several investigations have shown that miRNAs are mentioned in the osteoblast differentiation of stem cells. For example, miR-199a-5p controls the osteogenic induction of bone marrow mesenchymal stem cells by targeting TET2 [17]. In addition, miR-450b promotes osteoblast induction in vitro and strength bone generation in vivo by targeting the BMP3 signaling pathway [18]. Prior studies indicate that the downregulation of miR-125a-5p was detected during osteogenic differentiation of hADSCs by microarray profiles of small RNA molecules [19]. However, by far, the regulating mechanism of miR-125a-5p in the osteogenic differentiation of hADSCs has not been reported.

Therefore, the purpose of this research was to investigate the specific mechanisms of miR-125a-5p controlling osteogenesis in hADSCs under oxidative stress. It is expected that our study will provide a beneficial groundwork for the application of miR-125a-5p in bone restoration.

## 2. Materials and Methods

### 2.1. Isolation and Culture of Cells

The human adipose-derived mesenchymal stem cells were purchased from Cyagen, USA (No. HUXMD-01001). The hADSCs were cultured in Dulbecco's Modified Eagle Medium : Nutrient Mixture F-12 (DMEM/F12, Gibco) medium, which includes 10% fetal bovine serum (FBS, Sigma), 100 U/mL penicillin, and 100 *μ*g/mL streptomycin, cultured incubated in a 5% CO_2_ atmosphere at 37°C. The complete medium was changed in 48–72 h, and the cells were passaged when 70~90% confluence was reached. hADSCs were expanded until passage 3.

### 2.2. hADSC Osteogenic Differentiation

The cells (passage 3) were transplanted to 6-well cell culture plates. Cells were grown to 50–70% confluence in complete medium after 24–48 h [20]. The medium was completely replaced with an osteogenic differentiation medium, which consisted of human adipose-derived stem cell osteogenic differentiation basal medium, human adipose-derived stem cell osteogenic differentiation fetal bovine serum, penicillin-streptomycin, glutamine, ascorbate, *β*-glycerophosphate, and dexamethasone (Cyagen, USA). The cells were then cultured up to 21 days. The osteogenic medium was changed every 72 h.

### 2.3. Reactive Oxygen Species (ROS) Measurement

Cells were transplanted into 2 × 10^4^ cells/cm^2^ in 6-well plates. After 24 h, 50, 100, and 200 *μ*M H_2_O_2_ were added into the fresh medium to stimulate oxidative stress response, and cells were incubated for another 24 h. Then, the cells were incubated in a new solution without the FBS medium and including 10 *μ*M of the Reactive Oxygen Species Assay Kit (ROS Assay Kit, Beyotime, China) for 0.5 h. Finally, 1000 × *g* centrifugation of cells was conducted for 5 min and they were then washed with phosphate-buffered saline (PBS) for 2–3 times. The levels of dichlorofluorescein (DCF) were tested by flow cytometry (CytoFLEX, Beckman Coulter, USA) as prescribed by the manufacturer's instructions.

### 2.4. Quantitative Real-Time Polymerase Chain Reaction (qRT-PCR)

hADSCs were lysed and gathered using the TRIzol Reagent (Invitrogen, USA) according to the manufacturer's instructions. cDNA was synthesized using reverse transcription with a PrimeScript RT reagent kit (Takara, Dalian, China) and amplified using 2×SYBR Green qPCR Master Mix (TOYOBO, Japan) as prescribed by the manufacturer's instructions. Primers of human osteoblastic marker genes, miR-125a-5p, and U6 were compounded by RIBOBIO (Guangzhou, China). The primers for qRT-PCR are shown in Table 1. U6 and GAPDH were the reference genes of miRNA and mRNA, respectively. Relative expression levels were quantified by the 2^-*ΔΔ*CT^ method. Each experiment was repeated 3 times, and each assay was performed in triplicate.

### 2.5. Cell Counting Kit-8 (CCK-8) Assay

Cell viability was measured by the CCK-8 colorimetric assay. hADSCs (3 × 10^3^/well) were cultured to confluence on 96-well culture plates and incubated with increasing concentrations of H_2_O_2_ (50, 100, and 200 *μ*M) and increasing time (4, 6, 8, and 24 h). After treatment, the cells were incubated with 10 *μ*L CCK-8 for 0.5-4 h, and the absorbance was measured at 450 nm using a microplate reader (Sunrise, TECAN, Austria).

### 2.6. Western Blotting

The target protein productions were gathered in RIPA lysis buffer (Solarbio, China), including the protease inhibitor, and put on ice for 15 min. After processing through electrophoresis and membrane transferring, the membranes were incubated by the anti-VEGF (1 : 1000 dilution, ab32152, Abcam, Cambridge, UK) antibody. After washing, the membranes were incubated with the corresponding secondary antibody (Proteintech, Wuhan, China) as prescribed by the manufacturer's instructions. Protein bands were quantified using the ImageJ software (National Institutes of Health, Bethesda, MD).

### 2.7. Cell Transfection

After 24 h of seeding, the miR-125a-5p mimics and miR-125a-5p inhibitor were transfected into the cells by the Lipofectamine™ 2000 transfection reagent as prescribed by the manufacturer's instructions (Gibco Life Technologies, USA); miR-NC and inhibitor-NC were used as the control and negative control, respectively. The medium was replaced after 24 h, and the cells were gathered for subsequent experiments.

### 2.8. Alizarin Red Staining (ARS)

hADSCs or hADSCs transfected with miR-125a-5p mimic (50 nM), miR-125a-5p inhibitor (50 nM), and corresponding negative control were seeded into 24-well plates, respectively. After reaching 75–90% density, the hADSCs were induced using the osteogenic differentiation medium for a further 3 weeks. After 14 and 21 days, the hADSCs were fixed by the 4% Paraformaldehyde and stained with ARS for 15 min at 23 ± 2°C, followed by analysis under an inverted microscope (Leica DMI4000B, Leica, Germany).

### 2.9. Statistical Analysis

SPSS 19.0 software (IBM Corporation, Armonk, NY, USA) was used for the statistical analysis. Data are presented as the mean ± standard deviation (mean ± SD). Comparisons between groups were used one-way analysis of variance followed by Tukey's post hoc test. A *p* value < 0.05 was treated as significant.

## 3. Results

### 3.1. miR-125a-5p Inhibits Osteogenic Differentiation of hADSCs

**Figure 1:**
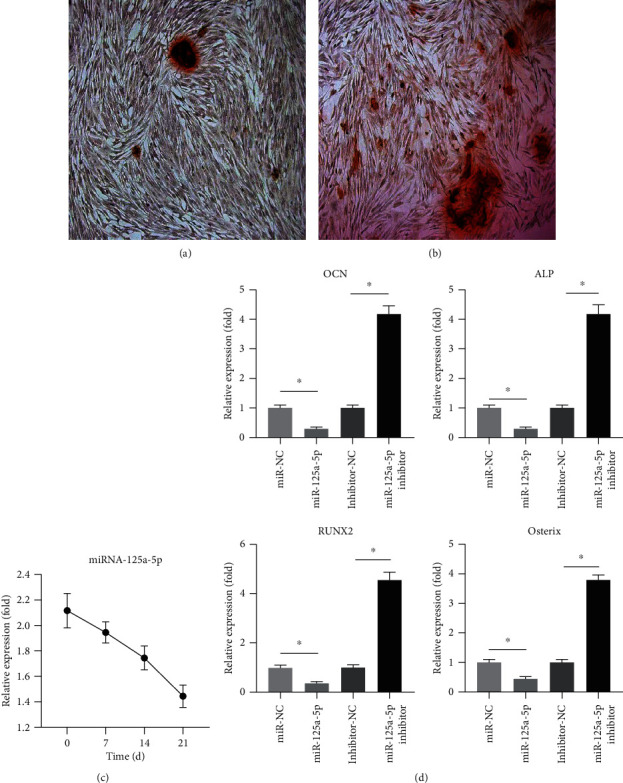
miR-125a-5p inhibits the osteogenic differentiation of hADSCs. (a, b) Osteogenic differentiation was induced in hADSCs on days 14 and 21 and was then processed with Alizarin red staining. (c) After the induction of osteogenic differentiation, the relative expression of miRNA-125a-5p was measured on days 0, 7, 14, and 21. (d) mRNA relative expression of the osteoblastic marker genes OCN, ALP, RUNX2, and osterix were measured after being transfected with miRNA-125a-5p and miRNA-125a-5p inhibitor. *N* = 3; ∗ means *p* < 0.05; ∗∗ means *p* < 0.01.

The presence of calcium deposition in hADSCs indicates that osteogenic differentiation was induced in hADSCs (Figures 1(a) and 1(b)). We first detected the expression level of miR-125a-5p in hADSCs by qRT-PCR in the osteogenic differentiation of hADSCs. The change of miR-125a-5p was reduced on day 7 compared with day 0 and continuously decreased to day 21 (Figure 1(c)). This suggested that miR-125a-5p might negatively regulate the osteogenic differentiation of hADSCs. hADSCs were transfected with the miR-125a-5p mimic and the miR-125a-5p inhibitor. The expression level of osteogenic marker genes was evaluated by qRT-PCR for osteogenic differentiation in 21 days. The results show that the mRNA level of the osteogenic marker genes was reduced after being transfected with the miR-125a-5p mimic, whereas the level expression of the osteogenic marker genes was increased after being transfected with the miR-125a-5p inhibitor (Figure 1(d)). It can be seen from these results that miR-125a-5p negatively regulated the osteogenic differentiation of hADSCs.

### 3.2. Effects of H_2_O_2_ on Cell Viability

**Figure 2:**
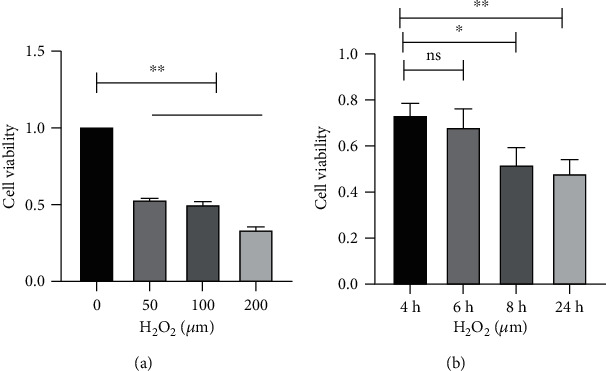
Effects of H_2_O_2_ on the viability of hADSCs. (a, b) Cell viability was measured by the CCK-8 colorimetric assay in different concentrations of H_2_O_2_ and induction times. *N* = 3; ^ns^*p* > 0.05; ^∗^*p* < 0.05; ^∗∗^*p* < 0.01.

The cytotoxic effect of H_2_O_2_ on hADSC viability was evaluated at 50, 100, and 200 *μ*M for 4, 6, 8, and 24 h using a CCK-8 assay. As shown in Figure 2, the cell viability was deceased by 50% with the 100 *μ*M H_2_O_2_ and for 24 h.

### 3.3. Oxidative Stress Induces Senescence of hADSCs and miR-125a-5p Expression

**Figure 3:**
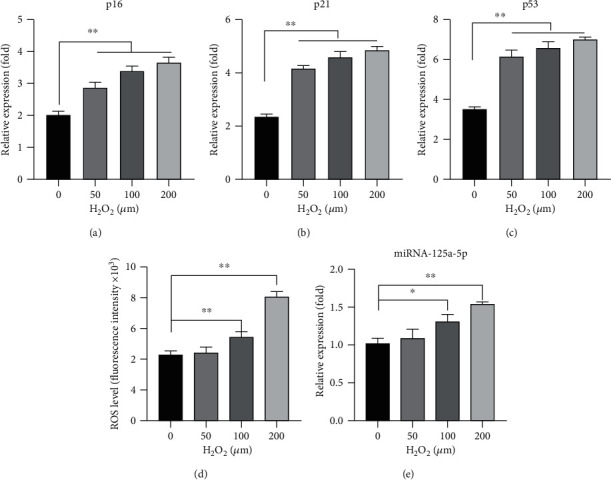
Oxidative stress induces senescence of hADSCs and miR-125a-5p expression. (a–c) mRNA expression levels of p16, p21, and p53 were evaluated by qRT-PCR. (d) The ROS level of the cells was measured by exposure to different concentrations of H_2_O_2_. (e) The expressions of miRNA-125a-5p were measured by exposure to different concentrations of H_2_O_2_. *N* = 3; ^∗^*p* < 0.05; ^∗∗^*p* < 0.01.

thADSCs were stimulated by different doses of H_2_O_2_ for 24 h to simulate the environment of oxidative stress response. As can be seen in Figures 3(a)–3(c), the three-cell senescence-related markers p16, p21, and p53 were significantly increased along with the dose of H_2_O_2_ and induction time. Furthermore, we can find that the level of ROS was increased along with the dose of H_2_O_2_, which was marked at 100 *μ*M and 200 *μ*M, and 100 *μ*M H_2_O_2_ was chosen for subsequent experiments (Figure 3(d)). The expression level of miR-125a-5p was increased following an increase in the concentration of H_2_O_2_ (Figure 3(e)). These data suggested that hADSCs exhibit an obvious increase in senescence with oxidative stress, which was the trigger to increase the expression level of miRNA-125a-5p.

### 3.4. Oxidative Stress Increases miR-125a-5p Expression during Osteogenic Differentiation of hADSCs

**Figure 4:**
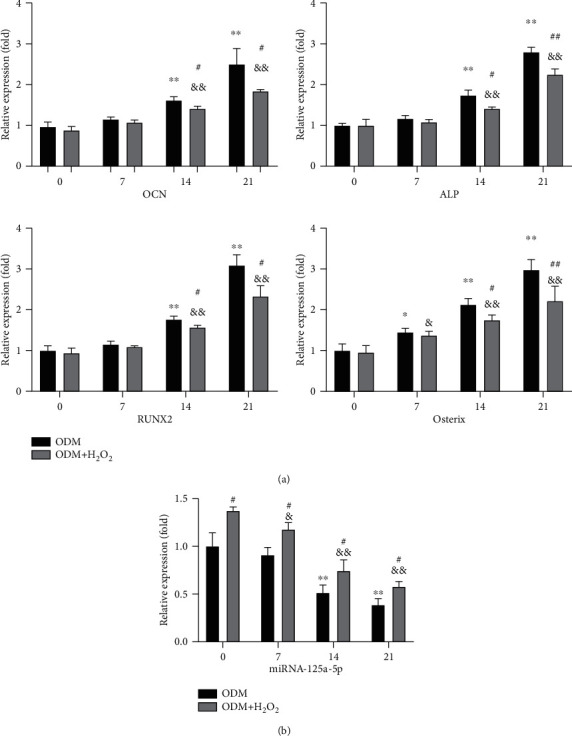
Oxidative stress increases miR-125a-5p expression during osteogenic differentiation of hADSCs. (a, b) The expression levels of the osteoblastic marker genes and the miRNA-125a-5p were measured with or without H_2_O_2_ in the condition of osteogenic differentiation medium (ODM). *N* = 3; ^∗^*p* < 0.05, ^∗∗^*p* < 0.01 vs. day 0 (ODM group); ^#^*p* < 0.05, ^##^*p* < 0.01 vs. day 0 (ODM+H_2_O_2_ group); ^&^*p* < 0.05, ^&&^*p* < 0.01 (ODM+H_2_O_2_ group vs. ODM group).

It was observed that the osteogenic marker genes continually increased when osteogenic differentiation was induced in hADSCs from day 0 to day 21. However, when osteogenic differentiation was induced in hADSCs in the presence of 100 *μ*M H_2_O_2_, the expression level of osteogenic marker genes decreased (Figure 4(a)). The level of miRNA-125a-5p was reduced in osteogenic differentiation, but the level in the medium with H_2_O_2_ was higher than that in the medium without H_2_O_2_ (Figure 4(b)). These results show that oxidative stress increased miR-125a-5p and concomitantly reduced osteogenic differentiation during the osteogenic differentiation of hADSCs.

### 3.5. miR-125a-5p Mediates the VEGF-Regulated Osteogenic Differentiation of hADSCs under Oxidative Stress

**Figure 5:**
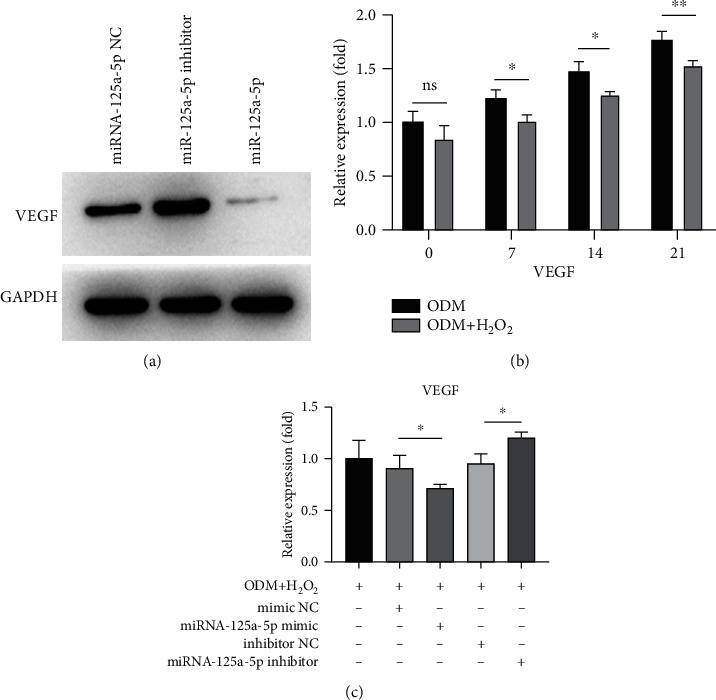
miR-125a-5p mediates the VEGF-regulated osteogenic differentiation of hADSCs under oxidative stress. (a) The expression level of VEGF protein in hADSCs infected by miRNA-125a-5p or infected with miRNA-125a-5p inhibitor was measured by western blotting. (b) The expression level of VEGF was evaluated at different time points during osteoinduction with or without H_2_O_2_. (c) The expression level of VEGF was measured by qRT-PCR on day 21, and after that, the cells were induced by the osteogenic differentiation medium (ODM) and, with the H_2_O_2_ stimulation, infected by miRNA-125a-5p or infected with miRNA-125a-5p inhibitor. *N* = 3; ^ns^*p* > 0.05; ^∗^*p* < 0.05.

By using the web-based bioinformatics tool, miRBase (http://mirtarbase.cuhk.edu.cn), a predictor gene is considered a target gene of miRNA when it met software's requirement. It was found that miR-125a-5p's regulated target gene is VEGF. Thus, we first analyzed VEGF levels in transfected cells and found that the VEGF proteins were significantly declined with transfected miR-125a-5p mimic and rose with transfected miR-125a-5p inhibitor (Figure 5(a)). Then, hADSCs were cultured under osteogenic conditions with 100 *μ*M H_2_O_2_, and the mRNA level of VEGF was evaluated by qRT-PCR at different time points during osteogenic differentiation. Although the mRNA level of VEGF showed marked increase during osteogenic differentiation up to 21 days, the VEGF levels were decreased when H_2_O_2_ stimulated the oxidative stress response (Figure 5(b)). Furthermore, under the conditions of oxidative stress, the overexpression of miRNA-125a-5p could suppress the expression of VEGF while the overexpression of miRNA-125a-5p inhibitor could promote the expression of VEGF (Figure 5(c)). These results provide strong evidence that miR-125a-5p could mediate the expression level of VEGF.

## 4. Discussion

ADSCs are found largely and can be obtained readily, and they have the ability to differentiate into numerous cell lineages, including hepatocytes, endothelial cells, smooth muscle cells, cardiomyocytes, neurons, adipocytes, osteocytes, and chondrocytes. Therefore, ADSCs are known as a tool for repairing, replacing, and regenerating damaged tissue, but the limited osteogenic differentiation ability greatly impedes the clinical application of ADMSCs in bone repair [21].

Specific regulatory factors and multiple signaling pathways are involved in the progression of osteogenesis [22, 23]. miRNAs have been shown to have a vital role in osteogenic differentiation and may become vital for regulating bone repair [24, 25]. The role of miR-125a-5p has been researched in a variety of cell lineages. As a functional microRNA, miR-125a-5p regulates the progression of bladder cancer by effecting on FUT4 [26]. Recently, a study has revealed that increased miR-125a-5p decreases the expression of TNFRSF1B and increases osteoclast differentiation [27]. The outcomes of previous studies suggest that miR-125a-5p affects the osteogenic differentiation of ADSCs [19]. This current study further explored the specific mechanism of miR-125a-5p in the osteogenic differentiation of hADSCs. We show an obvious decrease in miR-125a-5p expressions during the osteogenic differentiation of hADSCs. Meanwhile, the overexpression of miR-125a-5p suppressed osteogenic induction of hADSCs, and the inhibition of miR-125a-5p by transfecting inhibitor oligonucleotide increased osteogenesis. Taken together, these outcomes show that miR-125a-5p is a negative regulator of the osteogenic differentiation of hADSCs.

Senescence of MSCs can be caused by different stimuli, including oxidative stress, radiation, chemicals (inducing acute senescence), or replication failure (inducing chronic senescence) [28]. Oxidative stress is induced by an imbalance between an excessive reactive oxygen species (ROS) and lacking antioxidant defense mechanisms [29]. The properties and their linked differentiation of ADSCs are regulated by reactive oxygen species in some degree [30]. In this study, hADSCs were treated with 100 *μ*M H_2_O_2_ to mimic the environment of oxidative stress and induce the generation of ROS. When hADSCs were grown in osteogenic differentiation medium, the levels of osteogenic genes exhibited a time-dependent increase. However, the osteogenic genes tended to be reduced by oxidative stress stimulation. Meanwhile, under oxidative stress, ROS, the senescence of hADSCs, and miR-125a-5p were increased over time, suggesting that oxidative stress suppresses the osteogenic differentiation of hADSCs, induces the senescence of hADSCs, and is important for miR-125a-5p production.

Researchers have shown that VEGF has dual activities of angiogenesis and osteogenesis, which can significantly promote the growth of osteoblasts and accelerate bone formation [31]. Previous studies have also found that lamin A/C regulates the transcription factors Runx2 and PPAR*γ*2 by VEGF to control the osteogenic or adipogenic differentiation of mesenchymal stem cells. Moreover, the reduction of osteogenic differentiation of MSCs caused by VEGF gene deletion could only be restored by retrovirus-mediated VEGF expression, rather than by the exogenous recombination of VEGF [9]. In this study, it was proven using western blotting that miR-125a-5p controls the expression of VEGF in hADSCs. Meanwhile, we also showed that oxidative stress has a key role in regulating VEGF during the osteogenic differentiation of hADSCs. Oxidative stress and miR-125a-5p together suppressed the production of VEGF in the course of osteogenic differentiation of hADSCs, and the effect of oxidative stress could be reversed by the inhibition of miR-125a-5p.

**Figure 6:**
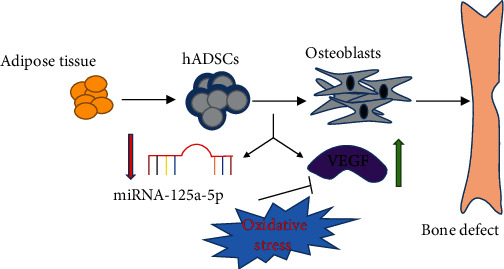
Schematic illustration of miRNA-125a-5p as it regulates osteogenic differentiation of hADSCs under oxidative stress by inhibiting the expression of VEGF.

In conclusion, our study pointed out that miR-125a-5p is induced under oxidative stress and inhibits the expression of VEGF, leading to the reduction of osteogenic differentiation of hADSCs (Figure 6). This demonstrates that miR-125a-5p could be a potential clinical target for bone repairing.

## Figures and Tables

**Table 1:** Primer sequences for qRT-PCR.

Gene or miRNA	Sequence(5′ to 3′)
miR-125a-5p	F: CGATTCCCTGAGACCCTTTAA
ALP	F: CGAGATACAAGCACTCCCACTTC
	R: CTGTTCAGCTCGTACTGCATGTC
Runx2	F: CAAGGACAGAGTCAGATTAC
	R: GTGGTAGAGTGGATGGAC
OCN	F: GGTGCAGCCTTTGTGTCCAAGC
	R: GTCAGCCAACTCGTCACAGTCC
Osterix	F: TGCTTGAGGAGGAAGTTC
	R: CTTTGCCCAGAGTTGTTG
p16	F: GATCCAGGTGGGTAGAAGGTC
	R: CCCCTGCAAACTTCGTCCT
p21	F: TGTCCGTCAGAACCCATGC
	R: AAAGTCGAAGTTCCATCGCTC
p53	F: CAGCACATGACGGAGGTTGT
	R: TCATCCAAATACTCCACACGC
GAPDH	F: CCATCTTCCAGGAGCGAGATC
	R: GCCTTCTCCATGGTGGTGAA
U6	F: CTCGCTTCGGCAGCACA
	R:AACGCTTCACGAATTTGCGT

## Data Availability

All experiment methods and the result generated or used during the study appear in the submitted article.
